# Variational autoencoder provides proof of concept that compressing CDT to extremely low-dimensional space retains its ability of distinguishing dementia

**DOI:** 10.1038/s41598-022-12024-8

**Published:** 2022-05-14

**Authors:** Sabyasachi Bandyopadhyay, Catherine Dion, David J. Libon, Catherine Price, Patrick Tighe, Parisa Rashidi

**Affiliations:** 1 J. Crayton Pruitt Family Department of Biomedical Engineering, University of Florida, Gainesville, USA; 2 Department of Clinical and Health Psychology, College of Public Health and Health Professions, University of Florida, Gainesville, USA; 3 Department of Geriatrics and Gerontology, Department of Psychology, New Jersey Institute for Successful Aging, School of Osteopathic Medicine, Rowan University, Glassboro, USA; 4 Department of Anesthesiology, College of Medicine, University of Florida, Gainesville, USA

**Keywords:** Computational biology and bioinformatics, Psychology, Diseases, Health care, Engineering, Mathematics and computing

## Abstract

The clock drawing test (CDT) is an inexpensive tool to screen for dementia. In this study, we examined if a variational autoencoder (VAE) with only two latent variables can capture and encode clock drawing anomalies from a large dataset of unannotated CDTs (n = 13,580) using self-supervised pre-training and use them to classify dementia CDTs (n = 18) from non-dementia CDTs (n = 20). The model was independently validated using a larger cohort consisting of 41 dementia and 50 non-dementia clocks. The classification model built with the parsimonious VAE latent space adequately classified dementia from non-dementia (0.78 area under receiver operating characteristics (AUROC) in the original test dataset and 0.77 AUROC in the secondary validation dataset). The VAE-identified atypical clock features were then reviewed by domain experts and compared with existing literature on clock drawing errors. This study shows that a very small number of latent variables are sufficient to encode important clock drawing anomalies that are predictive of dementia.

## Introduction

The clock drawing test provides a method for screening individuals for dementia. The clock drawing test consists of two parts: the command condition, where participants are required to “draw the face of a clock, put in all the numbers, and set the hands for *ten after eleven*”; followed by the copy test condition where participants are instructed to copy a model clock. Accurate clock drawing relies on various cognitive domains, and subtle changes in drawing can provide rich information about underlying cognitive functioning^1,2^. Command-based clock drawing requires comprehension for instructions, recalling the semantic attributes of a clock, working memory to process the linguistic components of the test instructions, effective mental planning, visuospatial processing, and motor skills to execute the drawing^3^. Although the copy condition also requires an array of cognitive function capabilities, it mainly relies on efficient visual scanning abilities, visuoconstruction, and executive functioning^4–6^. Literature also suggests that performance on the clock drawing test correlates with the Mini-Mental State Examination (MMSE) total score which is an alternative test of cognitive impairment^7,8^.

Several analog clock scoring systems have been proposed in the past^1,9,10^. These scoring systems range from nominal (right/wrong) to elaborate 22- or 31-point scoring^11^. Some are based on analysis of errors assessing semantics, graphomotor functioning, and executive control^1^. Different scoring protocols have been shown to have similar psychometric properties^12^. However, Spenciere et al. (2017) suggest that the subjective human component for interpreting clock drawing performance can yield different outcomes^13^. Furthermore, Price and colleagues found variance in intra and inter-rater reliability^14,15^. Variability in rater scoring introduces ambiguities that can potentially negatively impact the robustness of any diagnostic test based on the CDT.

Deep learning (DL) models could obviate this problem due to their ability to automatically extract high-level features from the data without any a priori feature engineering. Such high-level features are extracted in a data-driven manner by continuously assessing correlations between simpler features. The generality and predictive power of this nested hierarchy of features is only limited by the size of training data. DL models (given they have sufficient data to train), therefore, present an opportunity for developing objective scoring criteria for more robust clinical decision-making.

In this study, we developed an interpretable DL model to automatically learn key clock drawing features, and then validated this learning with “disease” classification in a sample of clinically diagnosed individuals with Alzheimer’s Disease (AD) or Vascular dementia (VaD) versus non-dementia peers. We used the final image output of the digital clock drawing test (dCDT)^16^ for this work. The dCDT uses digital pen technology with associated smart paper, which can record every pen stroke during drawing, allowing researchers to examine a multitude of clock drawing elements like latencies between pen strokes, graphomotor elements such as the size of the clock face and, the total number of pen strokes, etc.^5,17^. This technology, therefore provides additional benefits compared to the traditional clock drawing test because it can analyze the process by which the drawing was created rather than solely relying on the final created image. Automated scoring models developed on the dCDT in recent years^18–20^ have significantly outperformed traditional pen and paper clock drawing test. Their AUROC scores range from 0.89 to 0.93, compared against published AUROC scores from existing clinician scoring systems of 0.66–0.79^21^. In this study, the protocol of kinematic, time-based or latency, and visuospatial features extracted by the dCDT were not used. Rather, we used only the clock image or actual drawing produced by patients and research participants to train a DL model to screen dementia.

Despite its promise, many disadvantages plague the successful application of DL pipelines to medical image analysis. First, the number of parameters in powerful DL models are many orders of magnitude higher than in standard machine learning models such as logistic regression. Generally, these parameters are the network weights of a deep neural network (DNN), which allow the network to model complex, arbitrary input–output relationships. The use of a large parameter space begets the need for large, labeled datasets to train these DNNs. A DNN uses the error values defined as the distance between the predicted class probabilities and the ground truth to iteratively update its parameters. As a result, the final parameter values become entirely dependent on the dataset and typically fail to generalize to other classification tasks. In this work, due to availability of a large unlabeled dataset and a significantly smaller labeled dataset, we decided to use a semi-supervised DL model that can learn the intrinsic variations in clock images from the unlabeled data and use the small, labeled data to solve the classification task with minimal fine-tuning.

We used a variational autoencoder (VAE) model to perform the self-supervised learning task. VAE is a generative model which aims to learn a joint probability distribution over all variables present in a dataset^22,23^. This technique uses the accurate reconstruction of input images as an objective to learn a low-dimensional latent representation in the form of a pre-defined prior distribution. Deep generative models have been shown to improve classification accuracy in semi-supervised learning settings, especially when one has few labeled examples and many more unlabeled examples^24,25^. We used a large unlabeled dataset of clock drawings to train the VAE and a considerably smaller labeled dataset to subsequently fine-tune the trained VAE encoder network. The encoder represents the part of the VAE network that encodes a clock drawing into a low-dimensional latent space. This research is the first attempt at using semi-supervised DL models to analyze clock drawings. The primary aim of this project is to provide proof of principle that a modest number of features learnt from the raw CDT image can encode enough clock drawing anomalies to build an efficient dementia classifier. A secondary and concurrent aim of this project is to show that self-supervised pre-training using large, unlabeled clock drawing datasets can learn information-rich features that are capable of performing classification of dementia from non-dementia based on small amounts of labeled data.

## Results

### Participants

Table 1 describes the pertinent demographic characteristics of the participants who completed clock drawings for the training, initial classification and secondary validation datasets. Individuals in all datasets were above the age of 60. All participants in the classification cohort completed the clock drawing test to both conditions. In the training dataset, three participants could not draw the command clocks. Within the classification cohort, the dementia subgroup was significantly older, less educated and had lower MMSE total score on average compared to their non-dementia peers. In comparison to the training cohort, the classification cohort was predominantly comprised of white male participants.

**Table 1:** Demographics of cohorts.

Dataset	Number of samples	Mean age (S.D)	Mean education (S.D)	% of Female	% of Caucasian	Mean MMSE total score	Mean MoCA total score
Training clocks	13,580	73 (6)	13 (3)	49	85	26 (4.0)	N/A
Dementia	112	80 (6)	13 (3)	32	100	22 (2.6)	N/A
Controls	130	68 (6)	16 (2)	33	94	29 (1.1)*	25 (2.2)^†^

*S.D* standard deviation, *MMSE* Mini-Mental State Examination, *MoCA* Montreal Cognitive Assessment.

*This value is available only for 39 individuals in the Control cohort.

^†^This value is available only for 91 individuals in the Control cohort.

### Classification dataset (fine-tuning cohort)

**Figure 1:**
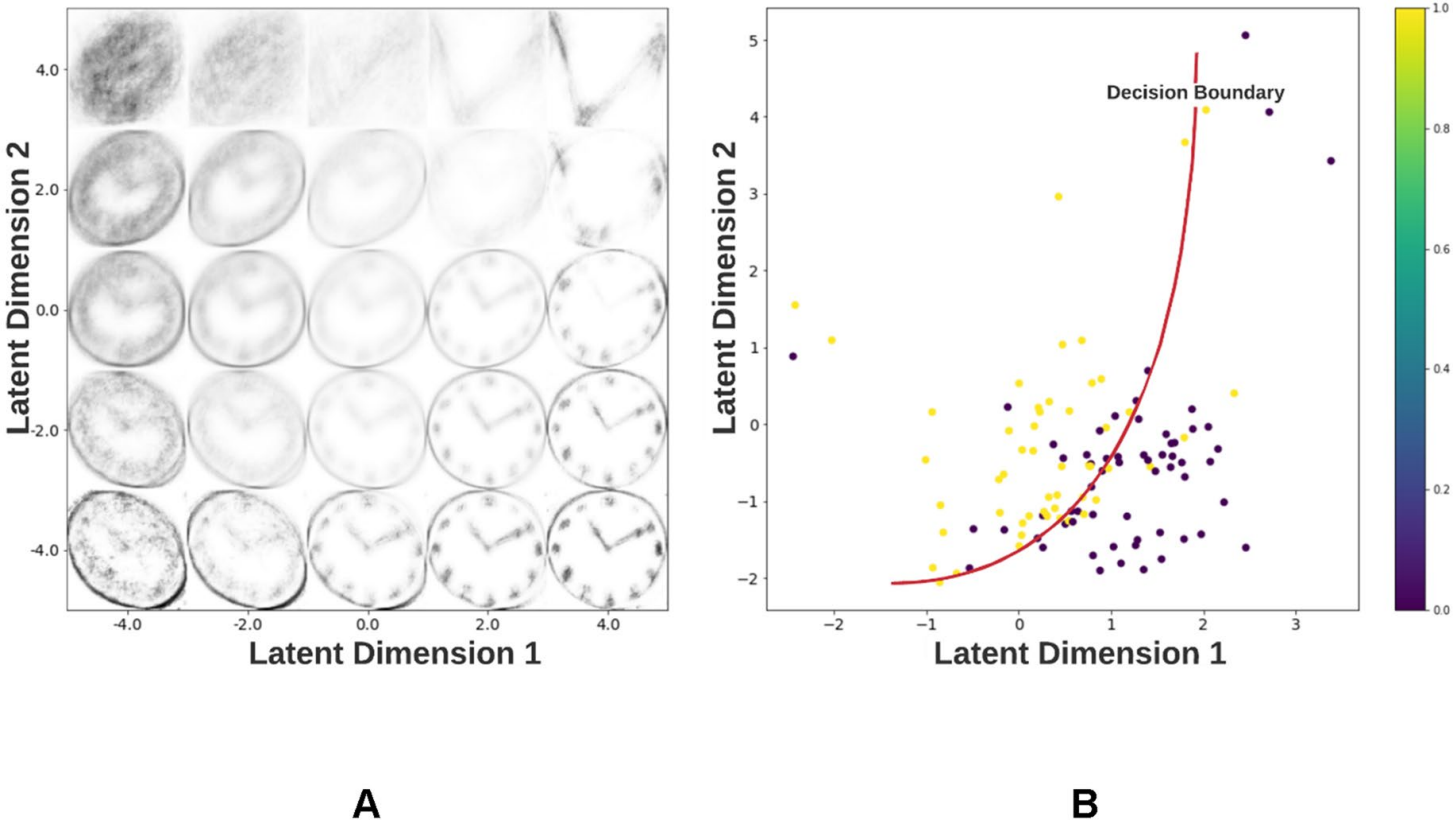
Distribution of reconstructed clocks in the VAE latent space. (**A**) Clock drawing reconstructions are represented as a function of the two VAE latent dimensions. This shows the variety of reconstructions generated by the VAE to capture the distribution of the training dataset. (**B**) Scatterplot showing the distribution of the latent vectors belonging to clocks in the fine-tuning dataset divided into dementia (= 1) and control (= 0) groups. The red curve represents a possible decision boundary between the two groups. Using our neural network classifier, we aim to learn such a decision boundary to classify the two groups.

Figure 1A illustrates the reconstructions of clock drawings performed by the VAE as a function of its latent variables. The smooth transition between the reconstructed clocks observed here results from the normal distribution to which the VAE latent variables are restricted. Figure 1B shows the distribution of reconstructions of dementia/control clocks from the fine-tuning dataset as a function of their latent space vectors.

**Figure 2:**
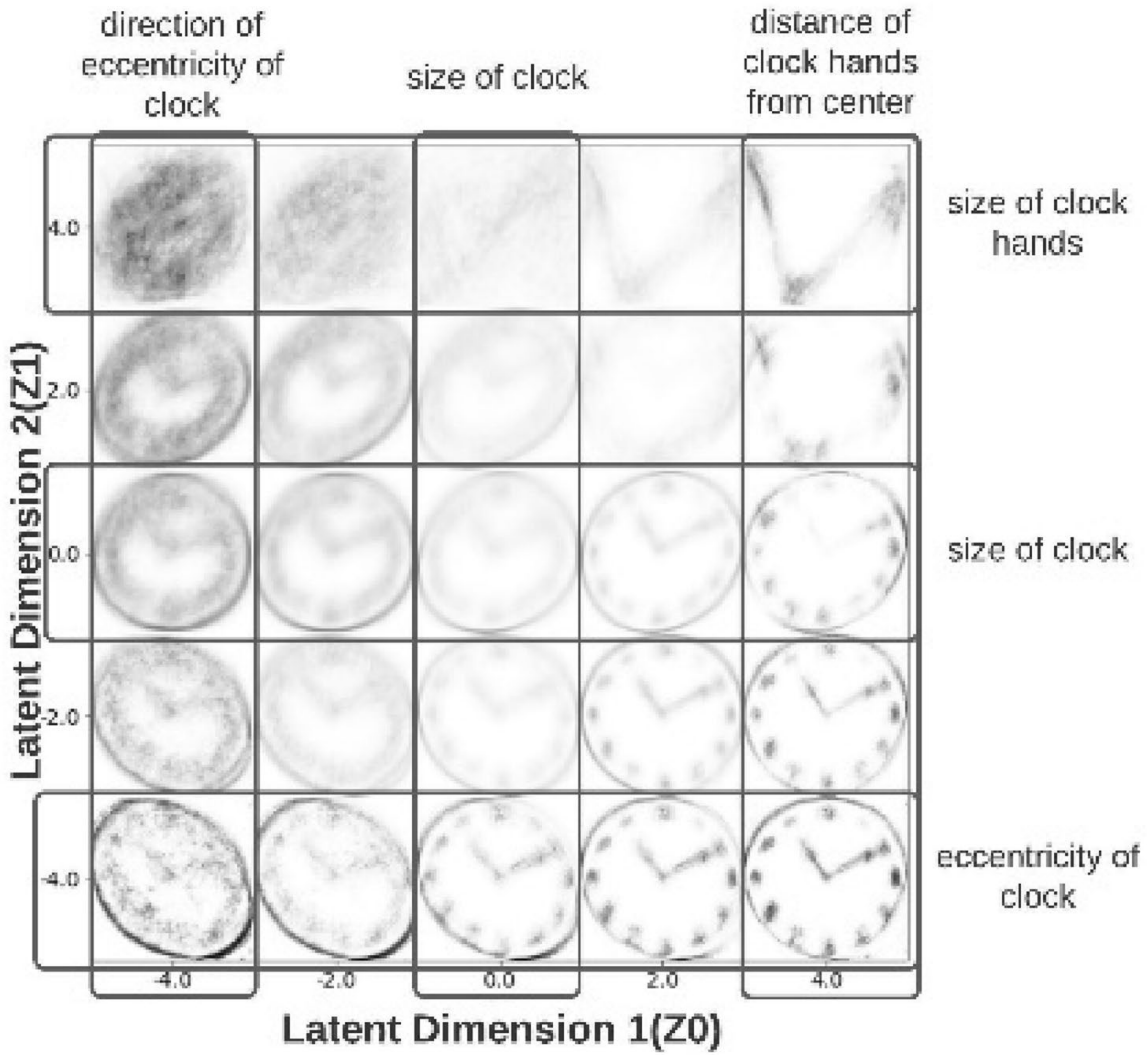
Generative factors of clock drawing are discovered using traversals over VAE latent space. Different regions of the two-dimensional manifold constructed by the VAE map to specific artistic features/anomalies of clock drawings. Latent Dimension 1 = Z0, Latent Dimension 2 = Z1. Left side of the VAE latent space: direction of eccentricity of reconstructed clocks reverses from left to right as Z1 increases from − 4 to + 4 given Z0 =  − 4. This change is correlated with a decrease in clock size shown by darkening of the reconstructed clock. Right side of the VAE latent space: distance of intersection point of clock hands from the geometrical clock center increases as Z1 changes from − 4 to + 4 given Z0 = 4. This change is correlated with a loss of the circular clock boundary. Top half of the VAE latent space: size of the clock hands increases as Z0 changes from − 4 to + 4 given Z1 = 4. This change is correlated with the loss of clock face boundary. Bottom half of the VAE latent space: angle of eccentricity of the clock decreases as Z0 changes from − 4 to + 4 with Z1 =  − 4. This change is correlated with an increase in clock size. X-axis of the VAE latent space: size of the clock increases as Z0 changes from − 4 to + 4 with Z1 = 0.

The scatterplot shows visible separation between dementia and control clocks. The reconstructions denote that features which are salient for human perception such as digits and ticks are not captured by the VAE latent space. Instead, the VAE latent space captures statistical features of clock drawings such as eccentricity, size, size of clock hands and distance of clock hands from the geometric center (Fig. 2). These features are correlated to the two latent dimensions of the VAE latent space and are mutually entangled in this space.

The latent manifold upon which the trained VAE projects clock drawings is a two-dimensional vector space that can be functionally divided into five regions which delineate the variation of different clock drawing features and anomalies. Figure 2 shows a traversal over these five regions. The VAE had no prior information about the generative features of clock drawing. Therefore, the clock features appear in an entangled/mutually correlated manner in the two-dimensional latent space of the VAE.

### Latent dimensions

The first latent dimension (plotted along the X-axis) is abbreviated as Z0, and the second latent dimension (plotted along the Y-axis) is abbreviated as Z1. The left half of the latent manifold space (Z0 < 0) is concerned with the direction of the eccentricity of a clock drawing (Fig. 2, Supplementary Video V1). The eccentricity reverses from left to right as Z1 given Z0 (Z1|Z0) changes from − 4 to + 4, passing through a point of zero eccentricity (circular clock at Z1|Z0 = 0). The size of the clock drawing is a correlated variable which decreases as Z1|Z0 increases from − 4 to + 4 in the left half of the two-dimensional manifold. On the other hand, the right half of the latent manifold (Z0 > 0) is related to the distance of the point of intersection of clock hands from the clock’s geometric center (Fig. 2, Supplementary Video V2).

The point of intersection of clock hands moves downwards from the geometric center as Z1|Z0 traverses from − 4 to + 4 in this region. This change is also associated with a loss of the circular periphery of the clock which is an important anomaly present in clocks drawn by patients with advanced stages of dementia. The top of the latent space (Z1 > 0) encodes an increasing length of clock hands; and distance of point of intersection of clock hands to geometric center mixed with each other (Fig. 2, Supplementary Video V3). Length of clock hands and area of the clock face increase as Z0|Z1 changes from − 4 to + 4. The bottom half of the latent space (Z1 < 0) encodes the eccentricity of the clockface (Fig. 2, Supplementary Video V4). Eccentricity decreases as Z0|Z1 changes from − 4 to + 4 in this region of the latent space. Furthermore, this feature is interlinked with an increase in clockface area as Z0|Z1 changes from − 4 to + 4 in this region. Finally, the X-axis which traces the change in Z0|(Z1 = 0) purely encodes the size of the clockface, evident from the increasing clarity of the clock drawing along this line (Fig. 2, Supplementary Video V5).

This analysis reveals that many physically understandable clock features and anomalies are encoded in different regions of the latent space of the trained VAE. It also shows that in many cases multiple physical features are entangled with each other.

### Classification dataset (testing cohort)

**Figure 3:**
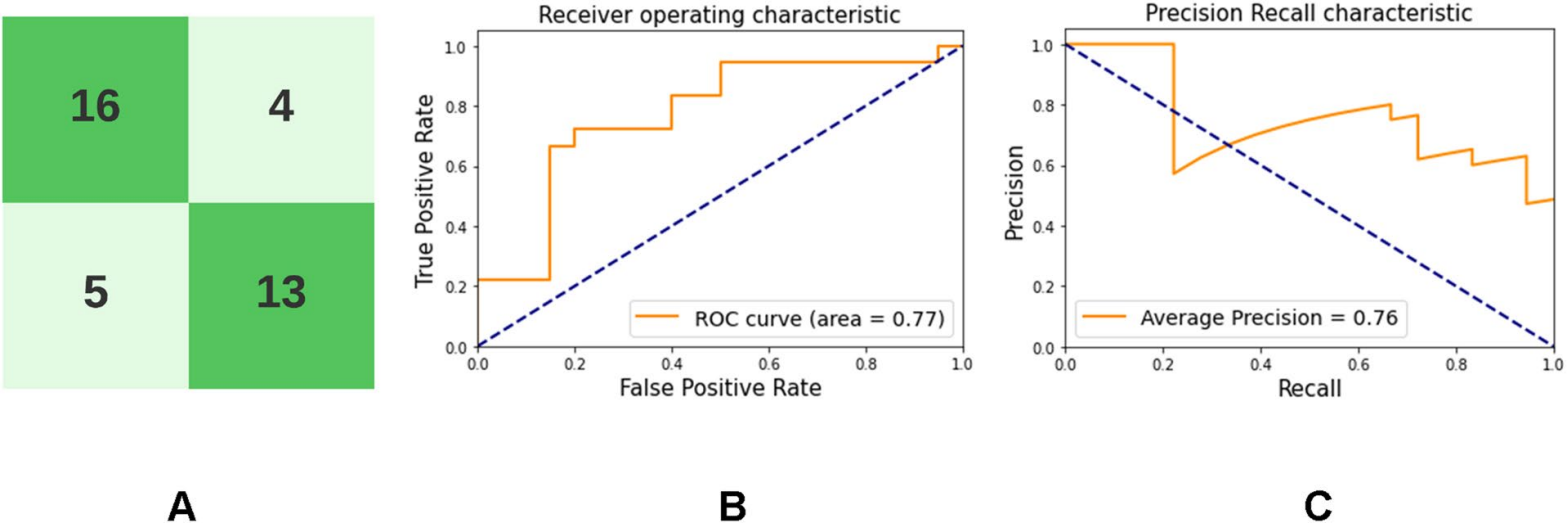
Performance of the classifier on test dataset. (**A**) The test dataset consists of 18 dementia and 20 control subjects. This image shows the confusion matrix. (**B**) Area under receiver operating characteristic is 0.77. (**C**) Area under precision-recall curve is 0.76.

The trained VAE encoder was used to perform classification after fine-tuning for 10 epochs over the fine-tuning dataset described previously. Latent space projections of the test dataset are shown in Supplementary Fig. S1. Table 2 shows the performance of the semi-supervised classifier constructed by employing the trained encoder network from the VAE. 95% confidence intervals denote the performance of the model over 100 bootstrapped examples of the test data. Figure 3 demonstrates the confusion matrix, receiver operating characteristic (ROC) curve and the precision-recall (PR) curve obtained upon classification on test data using this network.

**Table 2:** Performance of semi-supervised classifier on test data.

Dataset	AUROC (95% C.I.)	Accuracy (95% C.I.)	F1-score (95% C.I.)	Precision (95% C.I.)	Sensitivity (95% C.I.)	Specificity (95% C.I.)	NPV (95% C.I.)
Test	0.78 (0.59–0.91)	0.71 (0.54–0.83)	0.70 (0.51–0.82)	0.71 (0.47–0.87)	0.69 (0.49–0.86)	0.74 (0.54–0.87)	0.74 (0.49–0.88)
Secondary validation	0.77 (0.66–0.86)	0.75 (0.65–0.84)	0.74 (0.59–0.83)	0.73 (0.56–0.85)	0.75 (0.58–0.86)	0.76 (0.67–0.85)	0.79 (0.63–0.88)

*AUROC* area under the receiver operating curve, *C.I* confidence interval, *NPV* negative predictive value.

### Classification dataset (secondary validation cohort)

We further tested the classifier on a secondary validation dataset consisting of 41 dementia and 50 non-dementia clocks. Table 2 illustrates the model performance on this dataset. Here too, we employed bootstrapping with replacement to generate confidence intervals.

## Discussion

VAE trained on reconstructing digital clock drawings compress the relevant information present in a clock image into a highly informative 2-dimensional vector. The result appears to have useful properties for classifying dementia versus non-dementia. The latent space of the VAE can be used to generate artificial clock drawings which show statistical resemblance to the human-drawn clock dataset, but do not replicate salient features/details of clocks such as digits, hands, and ticks which are central to scoring clocks using traditional scoring techniques. Instead of local features, the trained VAE latent space captures global features such as clockface eccentricity, clockface area, length of hands and distance from the point of connection of hands to the clock center. Some of these features have been separately identified by domain experts as pertinent in distinguishing amongst various subtypes of dementia, as well as in separate cognitive tasks. For example, smaller clock face area is associated with micrographia and subcortical disease profiles where there is presence of primary executive dysfunction (e.g., Parkinson’s disease)^9^. Individuals with executive dysfunction and Parkinson’s disease also exhibit planning deficits in laying out numbers^9^. Clock face hand placement is also a consideration for disinhibition and visual attention difficulties^1^. The obfuscation of fine details of the clock drawing might be an inevitable trade-off related to the preprocessing step where clock drawings are resized to 100 × 100 to input them to the VAE. Future studies in this domain can find similar relationships between obliqueness of clock image or distance of the point of contact of clock hands from the center with underlying pathologies.

Furthermore, this analysis reveals that many physically understandable clock features and anomalies are encoded in different regions of the VAE latent space in a mutually entangled manner. In future work, we will investigate if disentangling these generative features improves the performance in dementia classification. To do this, we will use latent variable construction using advanced VAE models such as Factor-VAE^26–28^. In addition, we will investigate if a classifier built using the generative factors discovered in this paper can distinguish dementia from control. These generative factors are recorded in the software associated with the digital version of the clock drawing test that is used in this research^17^. We will augment these features with other graphomotor, and latency features solely available through the dCDT to examine the relative importance of these features in classifying dementia from nondementia.

The dCDT uses digital pen technology with associated smart paper, which record each pen stroke allowing researchers to access an array of variables inherent in the process by which the clock drawing was made in addition to the drawing itself. The dCDT can also capture full videos of the clock drawing test which can be analyzed using state of the art sequential deep learning models such as Vision Transformers^29^ to build high-resolution screening tools for dementia. These future research works will allow us to explore the wide range of data collection capabilities of the dCDT.

Importantly this methodology presents an important advance in bidirectional translational neuroscience involving AI. Here, we have used mechanistic understanding of dCDT tasks, developed in concert with neuroimaging studies, to train the latent representations of the VAE as part of a series of forward-translational experiments. Based upon visual inspection of the latent representations of the VAE, and in concert with the classification results, domain experts can use these findings to identify novel feature combinations of the CDT images and map these to gold standard cognitive assessments and/or neuroimaging findings. This bidirectional translational opportunity emphasizes the importance of methods which are sensitive to domain-level concerns including interpretability and mechanistic grounding.

**Figure 4:**
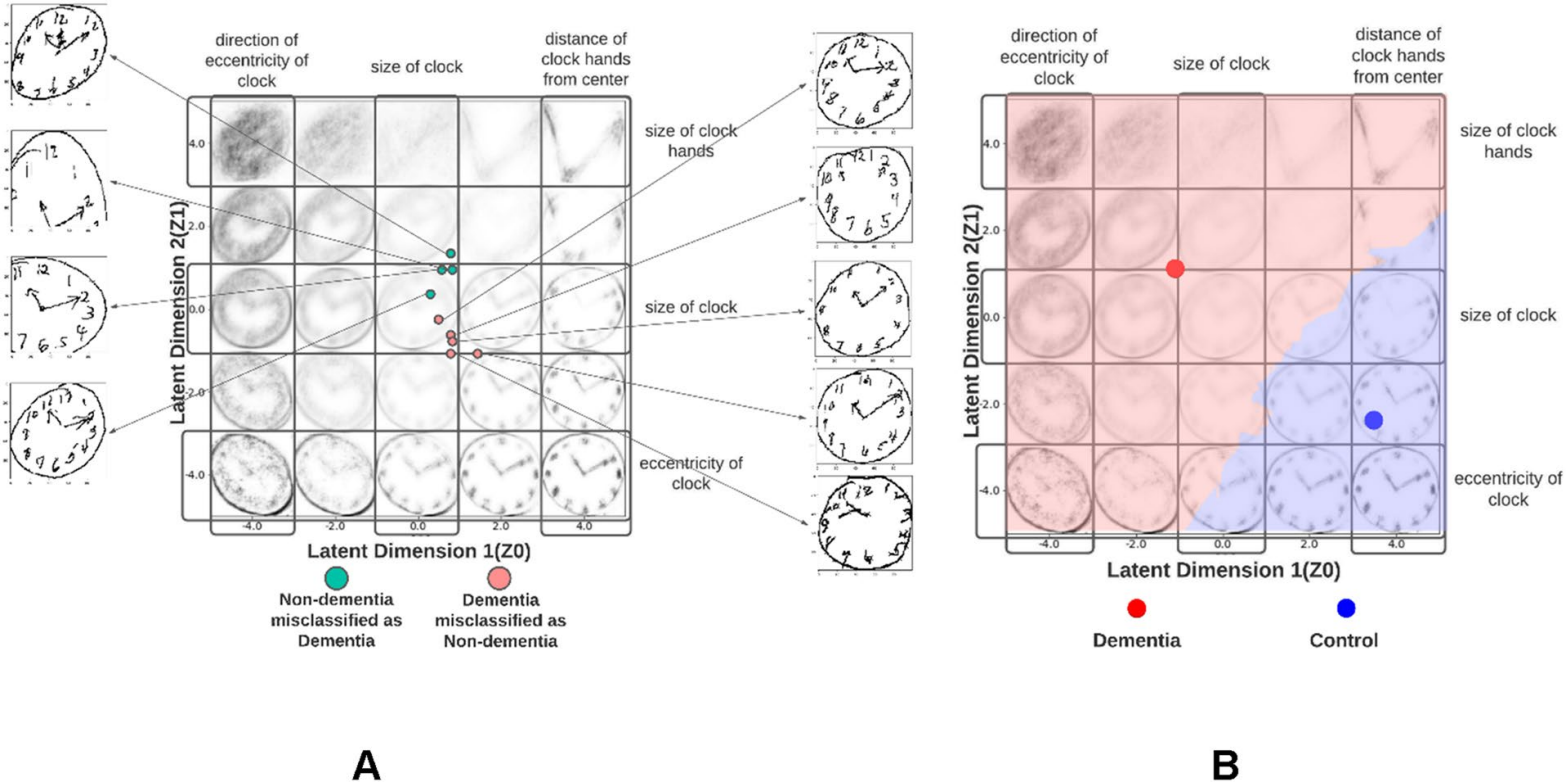
Increasing the interpretability and clinical utility of the VAE latent space. (**A**) Diagnosis of misclassified clock drawings in test dataset. The latent projections of misclassified clocks from the test dataset were compared to their original clock drawings (salmon represents dementia clocks misclassified as controls; green represents control clocks misclassified as dementia). Dementia and control clocks formed two distinct clusters in the latent space with the control clocks being projected towards the anomalous side of the latent space, thus justifying their classification. Mapping these projections to the original clock drawings revealed that our model correlated eccentricity to dementia and circularity to non-dementia. It failed to encode missingness/shortening of hands as a predictive feature variable in dementia, and our preprocessing pipeline mis-cropped certain clocks, thereby introducing eccentricity into them. (**B**) Operationalization of the VAE latent space using k-nearest neighbor classifier with k = 13. K-nearest neighbors of each training datapoint were labeled uniformly while simultaneously varying k to find the best decision boundary between dementia and control samples. This decision boundary physically divided the VAE latent space into two regions: Red—Dementia and Blue—Control. The control region is smaller, and it consists of normal sized clocks with circular clockfaces whose hands intersect at the geometric center of the clockface. The dementia region is larger in size and encodes the various anomalies detected by the VAE.

This study is not without its limitations. The dementia group is significantly older and less educated than the non-dementia group, thereby making the comparison unmatched. Furthermore, the objective of classification is more general than in other studies in this domain. However, this is acceptable since this study serves as a proof of principle that a two-dimensional representation of CDT retains the information required to classify dementia from non-dementia. We visualized the latent space projections of the 9 clocks from the test dataset which were misclassified to diagnose the potential shortfalls of the classifier (Fig. 4A). This revealed that- (a) misclassified dementia and non-dementia clocks formed two distinct clusters in the latent space where misclassified non-dementia clocks were projected towards the anomalous part of the latent space and vice versa, (b) misclassified dementia clocks had a normal size and circular clockface, but they were either missing hands or had shorter, misplaced hands and, c) misclassified non-dementia clocks either had eccentric clockfaces or were subjected to erroneous contour detection which produced partial clock-faces. This reveals two potential drawbacks of our model- (a) it could not encode absent/short, vertically displaced hands as a feature predictive of dementia, (b) during pre-processing, contour detection partially detected the clockface of some clocks. This introduced eccentricity in otherwise circular clockfaces. To improve the clinical utility and interpretability of our model we used a k-nearest neighbor classifier to operationalize the VAE latent space itself into “dementia” and “control” regions (k = 13) (Fig. 4B). The ideal k was decided using threefold cross validation on the fine-tuning dataset. This segmented the latent space into 2 regions corresponding to dementia and control. This classifier is different from the one reported in Table 1. The performance of this classifier is given in Supplementary Table T1.

In the future, we plan to use the compressed latent representation of CDT to differentiate between different types of dementia such as AD, VaD, mild cognitive impairment (MCI), amnestic-MCI, dysexecutive-MCI and Parkinson’s disease dementia. The encoding capability of the VAE was shown to depend on the amount of white space present in the sketches (Supplementary Fig. S2). This also limited our study as we had to discard the clock drawing images which were larger than 40,000 pixels in size.

In summary, this study demonstrated that the traditional pen and paper clock drawing test can be compressed into a parsimonious two-dimensional latent representation using contemporary semi-supervised DL models to classify participants diagnosed clinically with dementia. Since our classifier solely relies on the clock drawing and is trained in a weakly-supervised manner, it can potentially leverage large publicly available clock drawing datasets for creating similar classifiers with minimal fine-tuning for other conditions such as delirium and traumatic head injury. It can also be used to compare clock drawing images before and after a surgery or other insults. The same VAE encoder network, with minimal fine-tuning, can create new classifiers for different tasks in a short amount of time while using scant computational resources. Furthermore, such models can also be trained on the fly to monitor clock drawing changes over time, thereby serving as a simple, effective monitoring system of the neuro-cognitive health of an individual.

## Conclusion

In summary, in this study, we have shown that the CDT retains its discriminative potential even when reduced to a parsimonious two-dimensional space. Semi-supervised DL models trained on unlabeled clock datasets extracted generative clock features which are sufficiently informative to construct a dementia classifier. This is the first study using semi-supervised DL methods to analyze digital clock drawings. In the future, we will expand the classifier using graphomotor and latency features available from the dCDT and improve the latent space of the VAE using disentangled VAE in search for mapping between specific clock drawing errors and underlying cognitive pathologies.

## Methods

### Participants

Study materials were collected from digital clock drawing consortium data between the University of Florida (UF) and New Jersey Institute for Successful Aging (NJISA), Memory Assessment Program, School of Osteopathic Medicine, Rowan University. The Institutional Review Boards (University of Florida IRB and Rowan University, New Jersey Institute for Successful Aging IRB) approved this investigation, and permission was collected from the participants at both institutions through their signatures on informed consent forms. All study procedures were carried out in accordance with the Declaration of Helsinki and according to respective institution guidelines^30^. There were two data cohorts for the current investigation:

Training dataset -included a set of 13,580 clock drawings from participants age ≥ 65 years, primary English speaking, who completed clock drawing to command and copy conditions as part of routine medical care assessment in a preoperative setting^31^. Data were collected from January 2018 to December 2019. Exclusion criteria were as follows: non-fluent in the English language; education < 4 years; visual, hearing, or motor extremity limitation that potentially inhibits the production of a valid clock drawing.

Classification dataset—consists of *“fine tuning”, “test” and “secondary validation”* datasets with a set of individuals meeting criteria for dementia and a separate set of data from non-dementia peers. Dementia clocks are from individuals who had been evaluated as part of a community memory assessment program within Rowan University between February 2016 and March 2019. Individuals were seen by a neuropsychologist, a psychiatrist, and a social worker. Inclusion criteria: age ≥ 55. Exclusion criteria: head trauma, heart disease, or other major medical illness that can induce encephalopathy; major psychiatric disorders; documented learning disability; seizure disorder or other major neurological disorder; less than 6th-grade education, and history of substance abuse. All dementia participants were assessed with the Mini-Mental State Exam (MMSE), serum studies, and an MRI scan of the brain. Individuals have been described in prior research studies^32^. As described in prior scientific papers, these individuals were diagnosed with either AD or VaD using standard diagnostic criteria, respectively^33,34^.

Non-dementia peers had completed a research protocol of neuropsychological measures and neuroimaging with all data reviewed by two neuropsychologists. Inclusion criteria included age ≥ 60, English as the primary language, and availability of intact activities of daily living (ADLs) as per Lawton & Brody’s Activity of Daily Living Scale, completed by both the participant and their caregiver^35^. Exclusion criteria: clinical evidence of major neurocognitive disorder at baseline, as per the Diagnostic and Statistical Manual of Mental Disorders—Fifth Edition^36^, presence of a significant chronic medical condition, major psychiatric disorder, history of head trauma/neurodegenerative disease, documented learning disorder, epilepsy or other significant neurological illness, less than a 6th grade education, substance abuse in the past year, major cardiac disease, and chronic medical illness-induced encephalopathy. Participants were screened for dementia over the telephone using the Telephone Interview for Cognitive Status (TICS^37^); and during an in-person interview with a neuropsychologist and a trained research coordinator who also evaluated comorbidity rating^38^, anxiety, depression, ADLs, neuropsychological functioning, and digital clock drawing^39^. Data from these participants were collected from September 2012 to November 2019. These data have been described elsewhere^2,18^.

### Procedure

Cohort participants completed two clock drawings—one to a command condition and another to a copy condition^1^. The command condition required individuals to “Draw the face of a clock, put in all the numbers, and set the hands to ten after eleven”. The copy condition required individuals to draw the clock underneath a presented model of a clock. Drawings were completed using digital pen from Anoto, Inc. and associated smart paper^16,17^. The digital pen technology captures and measures pen positioning on smart paper 75 times/s. 8.5 × 11 inch smart paper was folded in half giving participants a drawing area of 8.5 × 5.5 inch for each clock. For the current project, only the final drawings were extracted and used for analyses.

Data from a training cohort of 13,580 unlabeled clock drawings to command and copy conditions were used to train the VAE in an unsupervised manner. Thereafter, the trained VAE encoder network, which compressed clock drawings into a low-dimensional latent space, was fine-tuned for distinguishing dementia from control clock drawings. Command and copy clocks were not separated at this stage as we wanted the model to extract features from clock drawings in a way that is agnostic to any cognitive outcome. This serves to keep the extracted features general and useful for any downstream classification task. The latent features extracted by the VAE were passed to a classification network for distinguishing dementia from control. This network was fine-tuned with 53 dementia and 60 control clock drawings, tested on 18 dementia and 20 control clock drawings, and further validated on another 41 dementia and 50 control clock drawings. A cohort diagram illustrating the process of creating these three datasets is shown in Supplementary Fig. S3. The fine-tuning and test datasets were created by randomly shuffling the classification dataset and splitting it in a 3:1 ratio. Therefore, there was no demographic difference between the fine-tuning and test datasets.

Individual clock sketches were extracted from file using contour detection. The extracted images were then flattened into one-dimensional vectors. These vectors were filtered to retain the ones with a size of less than 40,000 pixels (200 × 200). This filtering step was necessary to remove clock images containing excessive white space due to information sparsity issues. As clock drawings became sparser, the VAE tried to encode the interior white space of the clock instead of its drawn features, i.e., digits, hands, or circumference. This lead to poor latent space distributions that resembled white noise (Supplementary Fig. S2), high reconstruction errors, and ineffective VAE encoder weights. Therefore, we restricted clock sizes to a maximum of 200 × 200 pixels. The filtered clocks were then resized to a fixed size of 100 × 100 and converted into 1-dimensional vectors (10,000 X 1). These one-dimensional representations were used as inputs to the VAE. The preprocessing steps are illustrated in Supplementary Fig. S4. We replicated this preprocessing to extract 71 dementia and 80 control clocks from a total of 112 dementia and 350 control clocks that were initially collected using the exclusion criteria described above. They were divided into 53 dementia, 60 control clocks for fine-tuning the VAE encoder, and 18 dementia, 20 control clocks for testing it. However, we also used the remaining 41 dementia clocks and 50 randomly selected non-dementia clocks to prepare the secondary validation dataset as shown in Supplementary Fig. S3.

### Models and experimental setup

**Figure 5:**
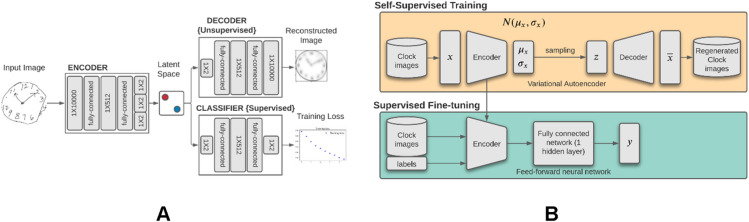
Conceptual workflow for training VAE with unlabeled dataset and constructing classifiers with latent space of VAE. (**A**) Clock images are passed into the VAE encoder in the form of a 1X10,000 vector. The top part of the schema represents the VAE decoder-encoder couplet which is trained to minimize the reconstruction loss of the clock drawing using an information bottleneck of a two-dimensional latent space. The lower part of the figure shows how the latent dimensions which capture a compressed representation of a clock drawing are passed into a classifier that mimics the architecture of the VAE decoder. This classifier is fine-tuned to reduce the loss in predicting dementia. (**B**) This image differentiates between the self-supervised pre-training of the VAE versus the usage of the pre-trained VAE encoder for downstream classification.

A variational autoencoder (VAE) is an unsupervised generative model with an encoding phase that projects input data onto a lower-dimensional latent space and a decoding phase that reconstructs the input data from random samples drawn from this latent space (Fig. 5). In the VAE model, the latent space distribution is created under the restriction that it follows a Gaussian distribution $N({Z}_{m}, {Z}_{s})$. This makes the VAE a generative model, as it can randomly sample this latent space distribution to create images resembling the input data which are not necessarily present in the input dataset. The use of a normal distribution as a prior does not lead to a loss of generality because the non-linear decoder network can mimic arbitrary input data distributions. No information about the generative features of clock drawing (e.g., total stroke length, clockface symmetry, coordinates of hands, number of digits, etc.) were supplied to the VAE network and we had no a priori expectations of making the latent dimensions represent these generative features. We used unidimensional representations of clock drawings to train the VAE with an input dimension of 10,000, an intermediate dimension of 512, and an embedding dimension of 2. The embedding dimension was intentionally kept extremely low to inquire if such a low-dimensional manifold can extract meaningful clock features useful for classification. The model was trained for 50 epochs with a batch size of 16. The reconstruction loss of the VAE was chosen to be the binary cross-entropy loss. The trained latent representation of the VAE was used as input to a feed-forward fully connected neural network with a single hidden layer of 512 neurons for classification studies. Figure 5 shows the architecture of our networks and a conceptual workflow of our method. The top portion of the figure shows the training of the VAE. The bottom portion of the figure shows how the compressed latent space of the VAE, in the form of trained encoder weights, was used to create a task-specific classifier. The classifier network had a fully connected feed-forward neural network architecture. We fine-tuned the weights of this classifier using a small, annotated fine-tuning dataset to improve its performance. The number of neurons in each layer of this classifier were finalized by using randomized grid search inside a threefold cross validation setting. The reported number of neurons gave the best average performance on the fine-tuning data over threefold cross-validation. Finally, the performance of this trained classifier was validated on the two validation datasets and several important performance metrics namely, AUROC, accuracy, sensitivity, specificity, precision and negative predictive value (NPV) were reported. The validation data were bootstrapped 100 times using random sampling with replacement to create confidence intervals of these metrics. The reported values constitute the median score, 2.5th quartile and 97.5th quartile of these metrics over the bootstrapped validation datasets.

## Supplementary Information

Supplementary Information.

## Data Availability

Datasets are available upon reasonable request. Reasonable requests will be reviewed to monitor compliance with the concerned authorities—National Institute of Health (NIH) and the Institutional Review Board (IRB). Relevant clinical trial numbers for the studies from which the datasets in this study have been constructed are NCT01986577 and NCT03175302.
