# Supplementary material for: Variational autoencoder provides proof of concept that compressing CDT to extremely low-dimensional space retains its ability of distinguishing dementia
Source: Sci Rep. 2022 May 14;12:7992. doi: 10.1038/s41598-022-12024-8 (PMC9107463; doi:10.1038/s41598-022-12024-8)
Supplement: Supplementary file 1 — Supplementary Information. [file 41598_2022_12024_MOESM1_ESM.docx]

**Supplementary Information**

Variational Autoencoder provides proof of concept that compressing CDT to extremely low-dimensional space retains its ability of distinguishing dementia.

Sabyasachi Bandyopadhyay, MS, Catherine Dion, MS, David J. Libon, PhD, Catherine Price, PhD, Patrick Tighe, MD, MS, Parisa Rashidi, PhD.

This supplementary material has been provided by the authors to give the readers additional information about their work.

**Supplementary Figures, Videos and Table**


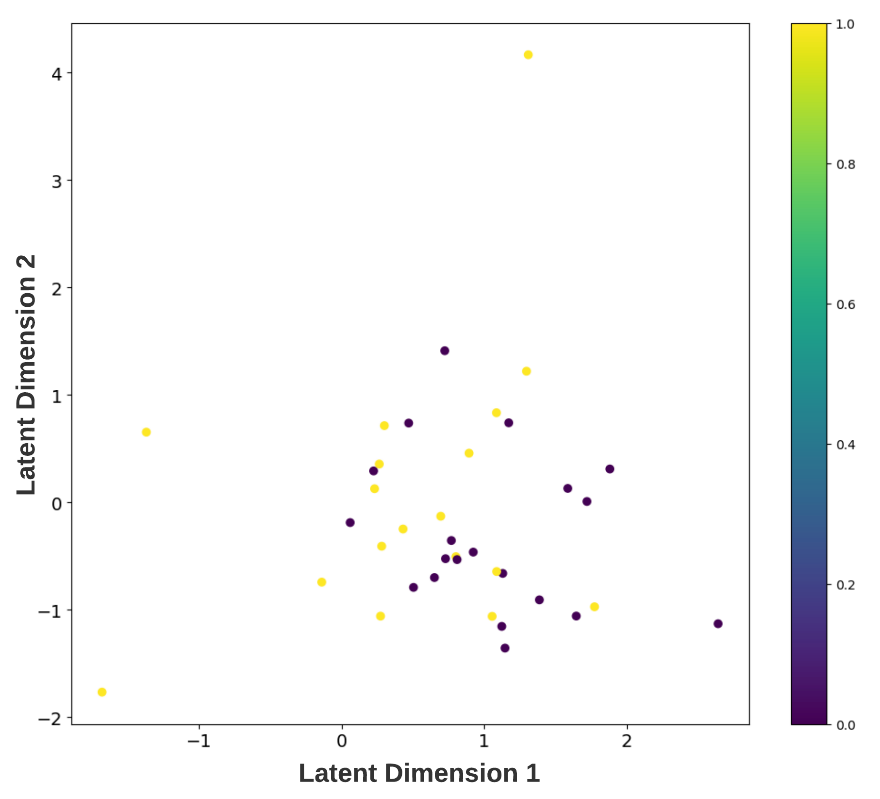


**Supplementary Fig S1. Latent space projections of clocks in the test dataset.** Scatterplot showing the distribution of the latent vectors belonging to clocks in the test dataset divided into dementia (=1) and control (=0) groups.


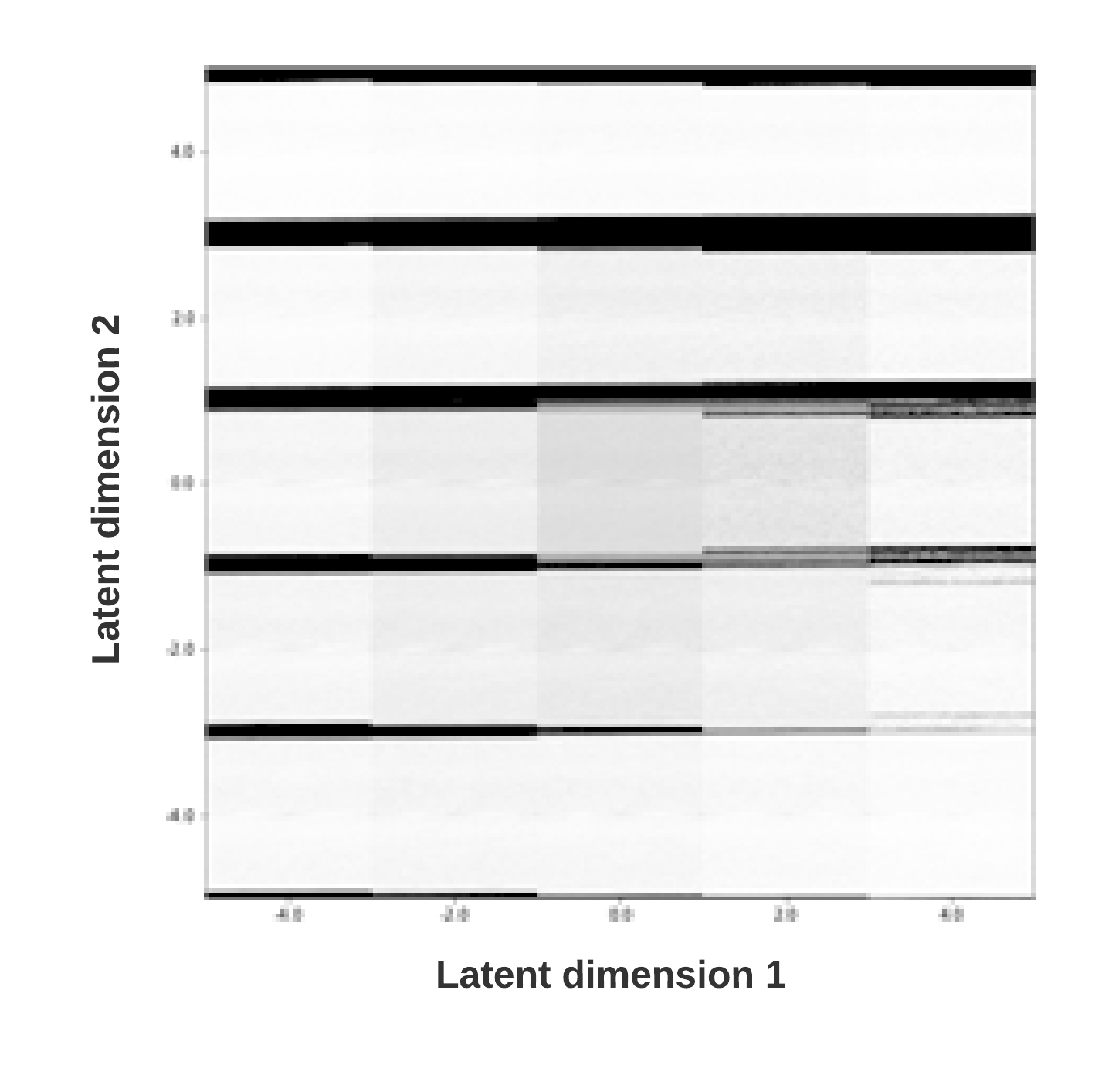


**Supplementary Fig S2. Clocks with excessive whitespace create VAE latent spaces which encode the whitespace inside clock drawings in the form of white noise instead of clock drawing features such as digits, hands and, clockface.** The VAE was initially trained on all clock drawings irrespective of their sizes. This resulted in an abnormal encoding of only the white space region inside the clock drawings onto the VAE latent space. The VAE understood that the closest it could get to statistically reconstructing a clock drawing was to encode its whitespace instead of the drawn features such as digits, hands and, clockface which are of clinical importance. This discovery led us to limit the size of clocks used in this study.


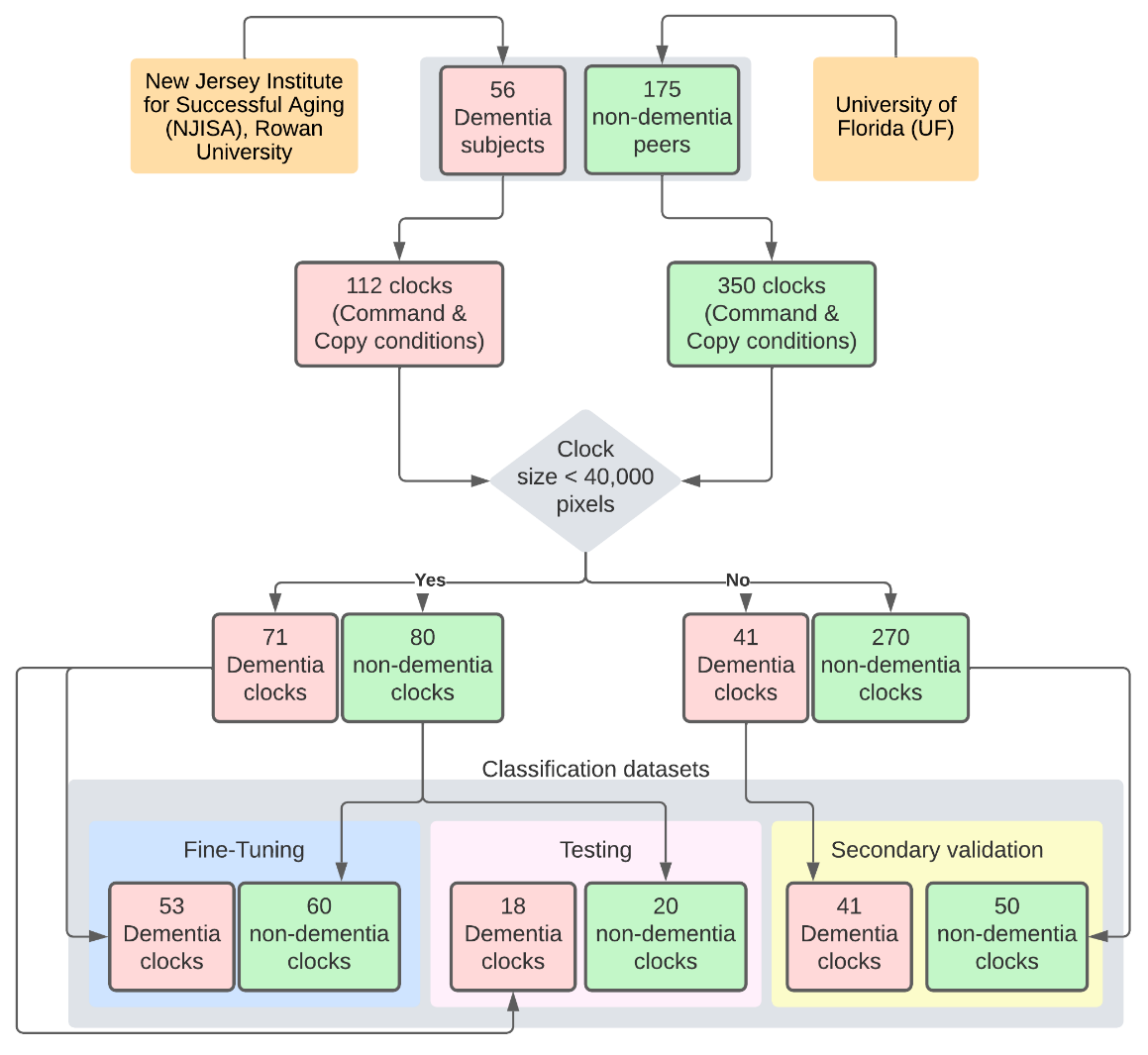


**Supplementary Fig S3. Cohort diagram for creating “fine-tuning”, “test”, and “secondary validation” subsets from classification dataset.** Rowan University and University of Florida provided clock drawing tests to both command and copy conditions of dementia subjects (total 112 clocks) and non-dementia peers (total 350 clocks). From these, 71 Dementia and 80 non-dementia clocks which were under 40,000 pixels in size were used to create the fine-tuning dataset (53 dementia clocks and 60 non-dementia clocks) and test dataset (18 dementia clocks and 20 non-dementia clocks). The remaining clocks which did not satisfy the size criteria were partly used to create a dataset of 41 dementia and 50 non-dementia clocks for secondary validation of the classifier.


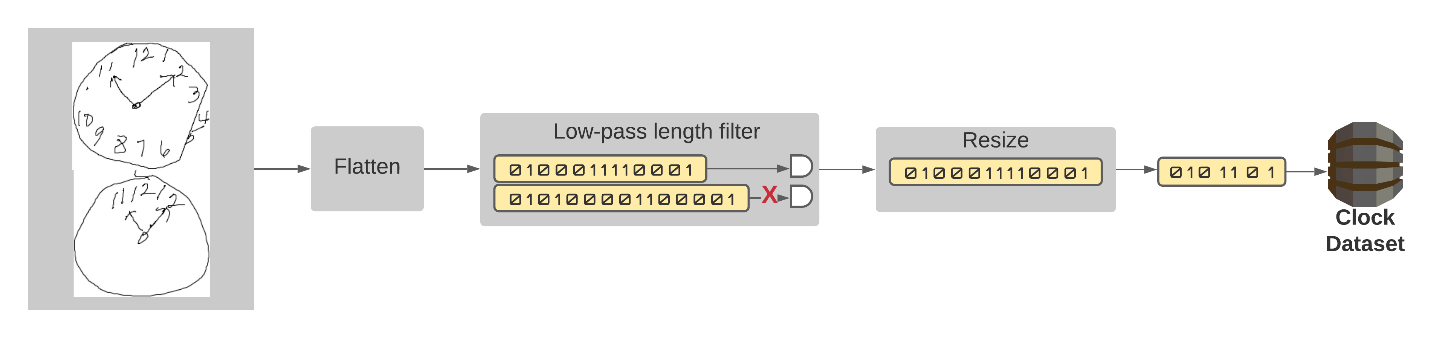


**Supplementary Fig S4. Preprocessing workflow of clock drawings.** Command and Copy clock drawings from a subject are flattened into 1-dimensional vectors. These are filtered to retain the ones which are less than or equal to 40,000 pixels. The resulting drawing vectors are then resized to 10,000 pixels and added to the training/fine-tuning datasets.


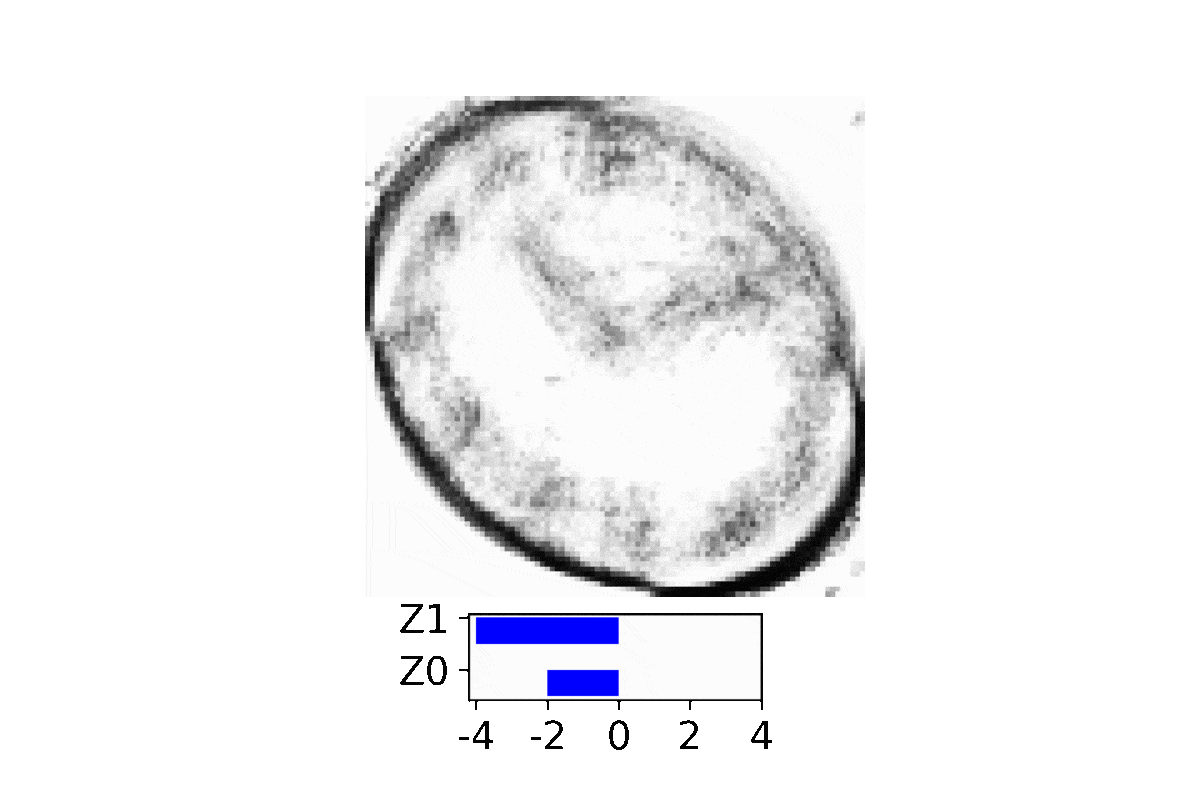


**Supplementary Video V1. Direction of eccentricity of clocks.** Direction of eccentricity of reconstructed clocks reverses from left at Z1= -4 given Z0 = -2 to right at Z1 = 4 given Z0 = -2. This change is correlated with a decrease in clock size shown by darkening of the reconstructed clock.


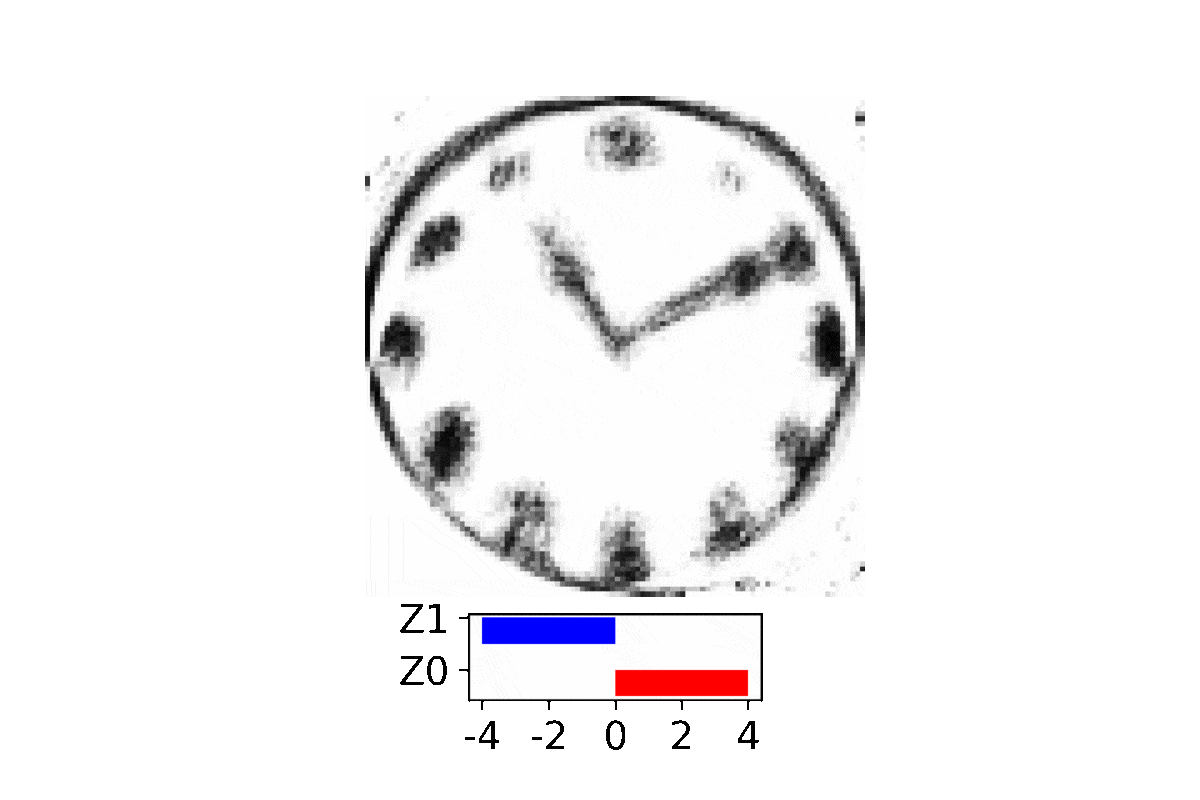


**Supplementary Video V2. Distance of point of intersection of clock hands from geometric center.** Distance of intersection point of clock hands from the geometrical clock center increases as Z1 changes from -4 to +4 given Z0 = 4. This change is correlated with a slight tilt of the clockface to the right and a loss of the circular clock face boundary.


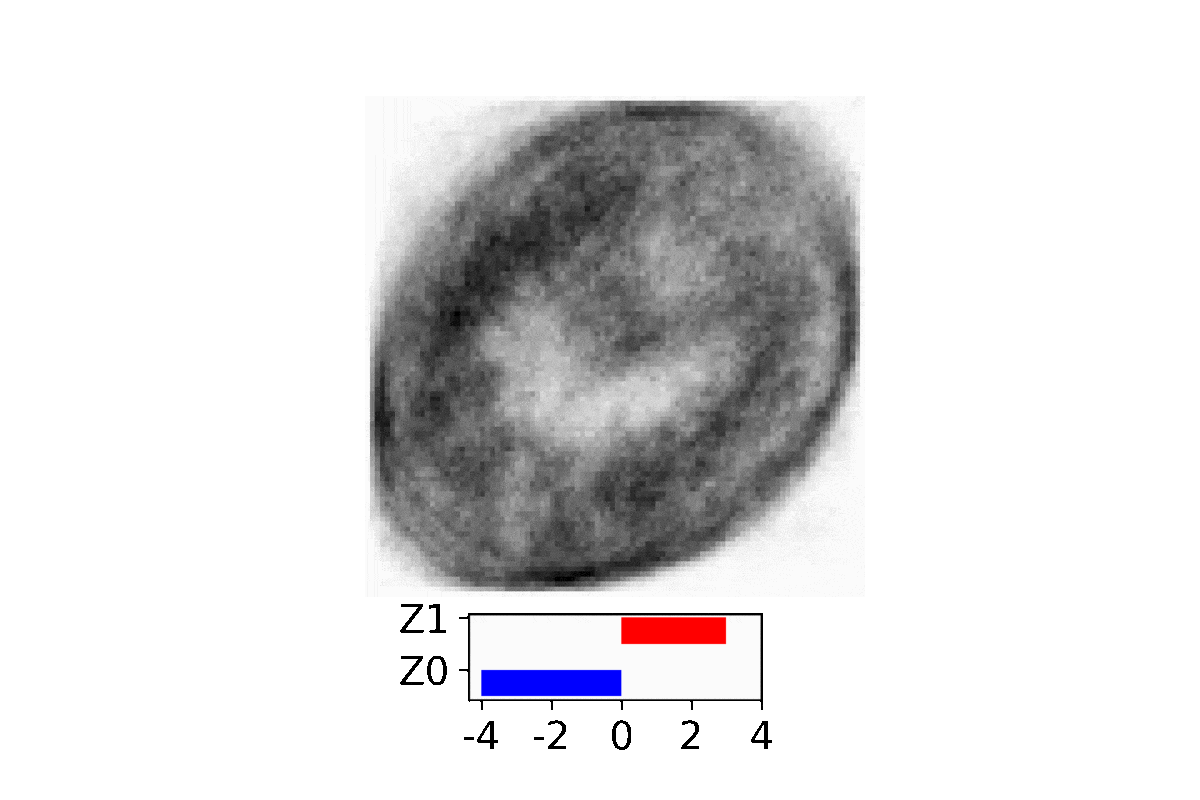


**Supplementary Video V3. Length of clock hands.** Size of the clock hands increases as Z0 changes from -4 to +4 with Z1 = 3. This change is correlated with a loss of the circular clock face boundary.


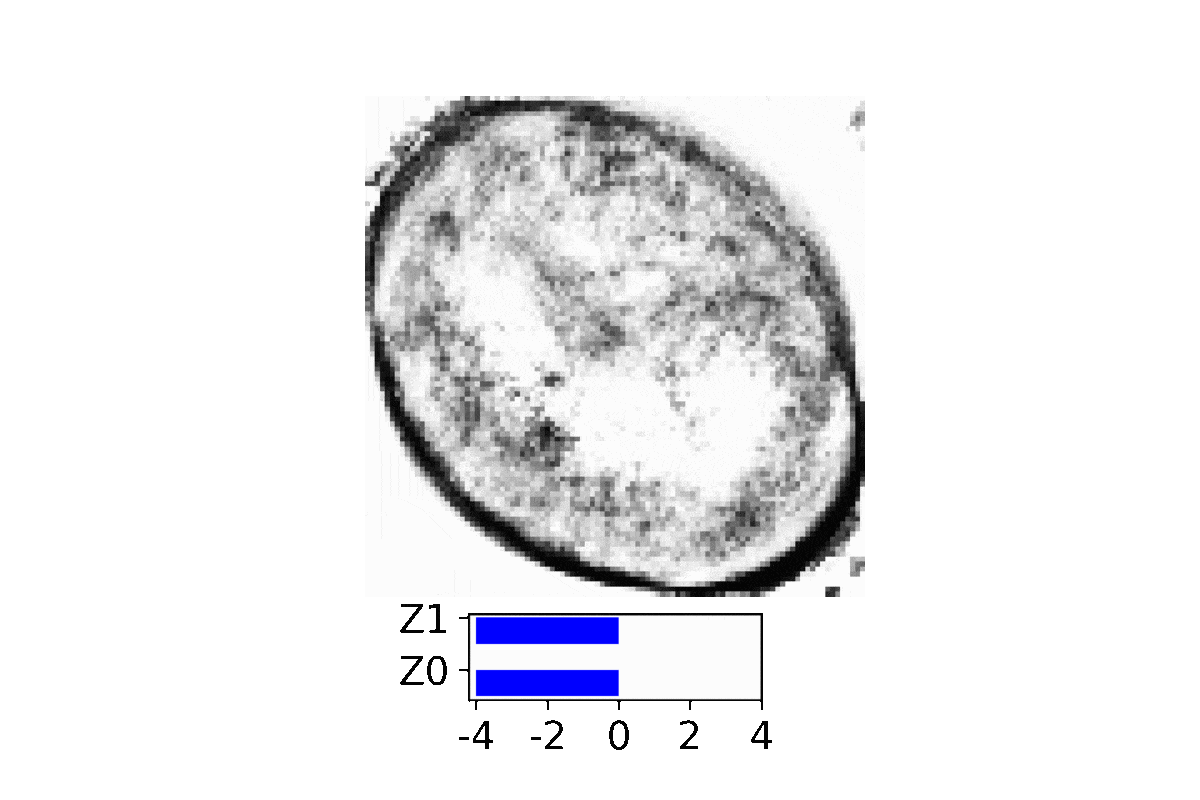


**Supplementary Video V4. Angle of eccentricity of clocks.** Angle of eccentricity of the clock decreases as Z0 changes from -4 to +4 with Z1 = -4. This change is correlated with an increase in clock size.


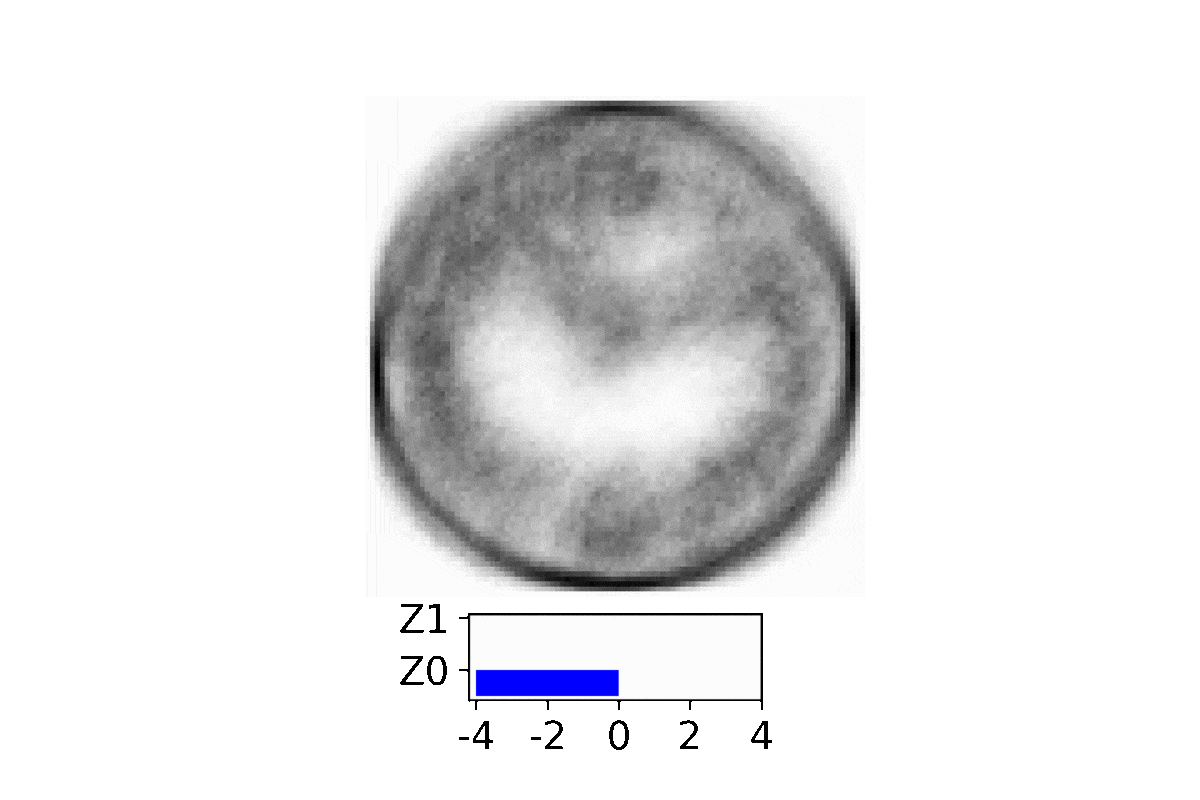


**Supplementary Video V5. Size of clocks.** Size of the clock increases as Z0 changes from -4 to +4 with Z1 = 0.

**Supplementary Table T1. Performance of k-nearest neighbor classifier on test dataset**

| Classifier | Dataset | AUROC (95% C.I.) | Accuracy (95% C.I.) | F1- score (95% C.I.) | Precision (95% C.I.) | Sensitivity (95% C.I.) | Specificity (95% C.I.) | NPV (95% C.I.) |
| --- | --- | --- | --- | --- | --- | --- | --- | --- |
| K-Nearest Neighbor (k=13) | Test | 0.78 (0.58 – 0.89) | 0.71 (0.55 – 0.87) | 0.71 (0.56 – 0.88) | 0.68 (0.50 – 0.92) | 0.78 (0.53 – 0.93) | 0.68 (0.46 – 0.92) | 0.77 (0.50 – 0.95) |

Abbreviations. AUROC, Area Under the Receiver Operating Curve; C.I, Confidence Interval; NPV, Negative Predictive Value.
